# The Impact of the Gut Microbiota on Humoral Immunity to Pathogens and Vaccination in Early Infancy

**DOI:** 10.1371/journal.ppat.1005997

**Published:** 2016-12-22

**Authors:** Quang N. Nguyen, Jonathon E. Himes, David R. Martinez, Sallie R. Permar

**Affiliations:** 1 Duke Human Vaccine Institute, Duke University School of Medicine, Durham, North Carolina, United States of America; 2 Department of Molecular Genetics and Microbiology, Duke University School of Medicine, Durham, North Carolina, United States of America; 3 Department of Pediatrics, Duke University School of Medicine, Durham, North Carolina, United States of America; 4 Department of Immunology, Duke University School of Medicine, Durham, North Carolina, United States of America; Geisel School of Medicine at Dartmouth, UNITED STATES

There is a new recognition that the gut microbiota, which consists of a dynamic community of intestinal bacteria, viruses, fungi, and archaea, can impact the development and function of humoral immunity and vaccine efficacy, opening the possibility of microbiome engineering for optimal immune response [1, 2]. In mouse studies, the gut microbiota was shown to increase the proportion of murine progenitor B cells in the gut lamina propria (LP), resulting in a greater antibody diversity due to recombination-activating gene (RAG) protein-mediated immunoglobulin (Ig) gene editing [3]. Furthermore, *Bacillus* spore surface molecules can stimulate intestinal B cells via IgM engagement in rabbits, suggesting that the intestinal bacteria can drive early B cell proliferation and survival in the gut-associated lymphoid tissue (GALT) [4]. Zeng and colleagues further demonstrated that gram-negative gut commensal bacteria can induce systemic IgG responses under homeostatic conditions in both humans and mice, and these antibodies may contribute to protection against *Escherichia coli* and *Salmonella enterica* infections [5]. Altogether, the gut microbiota appears to play an important role in activating the developing immune system and inducing protective antibody responses. On the other hand, the gut microbiota can suppress serum IgA and IgG responses in conventionally raised mice compared to germ-free mice, which may affect rotavirus vaccine efficacy [2]. Notably, structural mimicry of microbial proteins to a great number of immunogenic antigens prior to pathogenic infections has been recently recognized as an additional host factor in vaccine immunogenicity [6]. Therefore, a greater understanding of how various immune cells interact with the diverse gut microbiota may provide novel insights into the impact of the gut microbiota on host immunity.

In this review, we discuss the role of the gut microbiota in the development of host humoral immunity during early infancy and consider the possibility of the manipulation of gut microbiota to alter the quality of vaccine-elicited immune responses.

## How Might Gut Microbiota–Host Immune Cell Interactions Affect the Development of Humoral Immunity to Infection and Vaccines?

Emerging work deciphering the relationship between microbiome changes and pathogen-specific systemic and mucosal immune responses will provide more insights into the functional role of the gut microbiota in the development of host immunity. For example, HIV-infected peripheral blood contains similar bacterial elements to that of the intestinal microbiome, and IgA and IgG-expressing memory B cells from human terminal ileum are frequently polyreactive and primarily specific for commensal microbes and self-antigens, suggesting that the gut microbiota may prime mucosal antigen-specific B cell responses [7–9]. While HIV-associated gut inflammation has been associated with intestinal microbial dysbiosis and translocation, the extent to which the observed microbial dysbiosis is mediated by commensal organisms remains unclear. Interestingly, systemic antibody responses to gut commensal bacteria are not affected by enteropathy and B cell dysfunction during chronic HIV infection. The intestinal memory B and plasma cells primed in the gut microbiota, however, may home to other mucosal sites, including the mammary gland [10, 11]. The highly compartmentalized commensal-driven immune response control at specific mucosal sites may therefore contribute to the distinct roles of specific bacterial species in the humoral immunity to infections and the high microbiome variability across body sites [12–14].

The gut microbiota can also influence vaccine efficacy. For example, in both germ-free and antibiotic-treated toll-like receptor 5 (TLR5)^–/–^mice vaccinated with the trivalent inactivated influenza vaccine, low antigen-specific plasma cell frequencies and IgG concentrations were observed at 1 week post vaccination [15]. Interestingly, re-establishment of the gut microbiota restored the vaccine-specific IgG responses to those of pathogen-free mice [15]. Oral reconstitution with flagellated *E*. *coli* in germ-free and antibiotic-treated mice was also sufficient to restore TLR5-mediated stimulation of activated B cell differentiation into plasma cells and enhance the magnitude of virus-specific antibody responses [15]. In contrast, the gut microbiota can adversely affect vaccine efficacy by skewing antibody responses towards distractive nonprotective vaccine antigens that resemble commensal bacterial antigens [2]. For example, pre-existing memory T and B cells specific to HIV-1 envelope (Env) glycoprotein that cross-react with gut commensal antigens may direct the antibody response to nonprotective gp41-directed epitopes that decrease HIV vaccine efficacy [16, 17]. In the setting of HIV-1 infection, having pre-existing gut microbiota-reactive IgG responses may adversely affect the development of functional HIV Env-specific neutralizing antibodies. Thus, altering the existing gut microbiota or the development of the microbiome in infants before memory B cells are fully initialized in the developing immune system may have a beneficial impact on B cell priming towards more functional antibody responses both in the setting of natural HIV infection and vaccination.

## When Is the Ideal Window for Gut Microbiota Manipulation to Optimize the Humoral Immunity in Early Infancy?

Gut microbial colonization in infants may begin in utero as bacteria from the placenta and amniotic fluid colonize the fetal intestinal epithelia [18]. Although the presence of an antepartum gut microbiota and its potential contribution to the gut-associated Ig diversification during human embryonic development and infancy demand further investigation, pre-B cells have been found in human fetal gut LP [3]. Moreover, enteromammary trafficking of maternal gut microbiome has also been proposed as a strategy for infant gastrointestinal (GI) tract colonization, involving intestinal bacteria carried by leukocytes from the maternal GI tract to the mammary glands via systemic circulation [19]. Furthermore, changes in polysaccharide-degrading gene expression levels in *Bifidobacteria* and *Bacteroidetes* in humans correspond to a change in diet from breast milk to solid food around 4–6 months of life, suggesting a functional maturation of the infant gut microbiota during the weaning period [20, 21]. The early B cell repertoire in neonatal mice, which primarily consists of maternal antibodies and immature or naïve B cells that have not undergone somatic mutation, also continues to develop and contribute to the long-lived B cell repertoire until around the weaning age [22]. Interestingly, the gut microbial exposure in the first 2 months of life in newborn rabbits and at up to 6 months of age in chickens may contribute to the specificity of early B cell repertoire in the GALT [3]. In fact, B cell receptor editing process in murine gut LP begins at the weaning age [3, 22].

**Fig 1:**
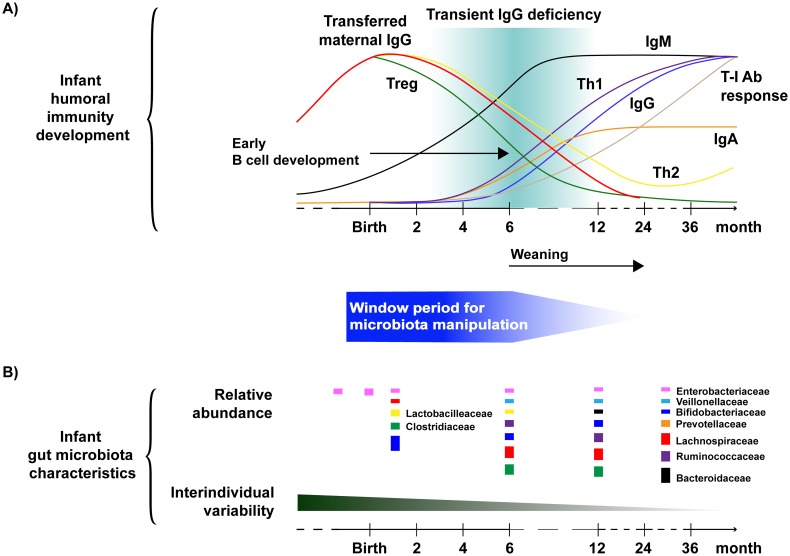
Early infancy as a window of time to modify the gut microbiota to support effective vaccine responses. Defining a critical time window for the manipulation of gut microbiota to support effective immune and vaccine responses may involve further investigation of (A) the infant humoral immune response development kinetics and (B) the characteristics of infant gut microbial colonization. (A) Curves represent various T and B cell response levels in newborn infants, with the upper bound being 100% of the adult levels. Kinetics of antibody responses were adapted from *Janeway’s Immunobiology* [28], Martin et al. [27], and Simon et al. [26]. The most abundant bacterial families of an infant gut microbiota at certain time points are shown with the size of the boxes representing their relative proportions. The relative abundance of bacterial families was based on studies by Arrieta et al. [36] and Collado et al. [18]. Abbreviations: T-I Ab response, T-independent antibody response.

Other immune development factors during early infancy, such as the presence of maternal antibodies and intestinal gut microbiota-primed B cell trafficking, may also be important considerations for the timing of the window period for gut microbiota manipulation [20, 23]. While maternal antibodies confer passive immunity during the first year of life, high maternal antibody titers may suppress infant vaccine-elicited immune responses through epitope masking and vaccine inhibition [24, 25]. As the level of passively acquired maternal IgG gradually decreases in the first year of life and infant IgG production increases over the first 6 months, the optimization of potential gut microbiota-directed infant immunization should consider passively acquired maternal antibodies [26–28]. Additionally, the gut microbiota may also modulate the recruitment of both IgM and IgA-secreting B cells as well as innate-like natural B1 cells to the gut LP [29]. Given the major role of the infant gut microbiome in early B cell development at or around the weaning age, an “intervening window” in which the gut microbiome can be manipulated may include birth and extend up to the late infancy or weaning period (Fig 1).

## What Factors Can Shape the Functional Composition of the Gut Microbiota to Optimize Immune Responses during Early Infancy?

A more defined timeframe of the gut microbiota-driven diversification of early B cells during infancy demands further understanding of the relative impact of various factors on the healthy development of the gut microbiota and humoral immunity [20]. For instance, prematurity has been shown to influence the progression of bacterial colonization of infant gut more than antibiotic exposure, diet, or birth mode [19]. Yet, birth mode, antibiotic usage, and diet may affect the timing but not the order of microbial succession in preterm infants [30]. In infants born at term, exclusive breastfeeding—rather than early introduction of solid food—has been shown to greatly influence initial gut bacterial colonization and enhance the postnatal maturation of the gut microbiota and subsequent infant immune system development during the first year of life [1, 20]. Similarly, while cesarean section-delivered infants are more likely to be exposed to antibiotics in utero than vaginally delivered infants, preterm infants are more likely to be formula fed and exposed to antibiotics beginning in utero [31].

Remarkably, recent work has demonstrated that breastfed infant rhesus monkeys have less fluctuation in their gut microbiota composition that is associated with a more robust development of T helper 17 (T_H_17) cells at 1 year of age as compared to bottle-fed infants [32]. The search for specific breast milk factors that modulate gut microbial colonization stability and T_H_17 development in humans could reveal interventional strategies to improve vaccine or other immune-based therapeutic responses. Hence, the impact of the gut microbiome on the helper T cell kinetics of T_H_1/T_H_2 cytokine-biased responses, regulatory T cells (T_regs_), and natural killer (NK) cell frequency, as well as IgG subclass composition, during infancy should also be examined to further define the period for an infant microbiota-directed vaccination strategy (Fig 1A) [26]. Furthermore, we are just beginning to appreciate the role of specific bacterial species in modulating immune responses. For instance, the relative abundance of certain commensal infant gut bacterial species, such as *Bifidobacterium*, may enhance oral vaccine efficacy, whereas others may lower vaccine-elicited antibody responses in infants [33, 34]. Thus, future microbial manipulation efforts that target the major groups of the gut micriobiota, especially before the the first 6 months of age—e.g., probiotic supplements, changes in diet and the current vaccination strategy—may be valuable for engineering an optimal vaccine-elicited immunity (Fig 1B).

## How Should Current Knowledge of the Interactions between the Gut Microbiota and Infant Immune System Guide Future Research and Novel Infant Vaccination Strategies?

Although the effect of various factors that can influence the infant gut microbiota can take place in utero, after birth, and into childhood, deeper knowledge of these factors on the microbiota–immune cell interactome during infancy should better refine the design of potential therapeutic intervention. With the recent advancement in high-throughput sequencing and gene-editing technology, functional changes in the gut microbiome can now be tracked with unprecedented resolution. Given the dynamic nature of the infant gut microbiota and the importance of robust humoral immunity to protection against life-threatening pathogens, future pediatric vaccine research should evaluate the manipulation of the infant microbiome to modulate desirable immune responses. Not only are the developing immune systems of infants distinct from those in adults, but the composition and influence of the gut microbiome on infant immune system is also unique [35]. Therefore, the infant microbiome should be explored as potential source of immune response modulation and enhancement of vaccine efficacy.
